# Leveraging flexible pipette-based tool changes to transform liquid handling systems into dual-function sample preparation and imaging platforms

**DOI:** 10.1016/j.ohx.2025.e00653

**Published:** 2025-05-02

**Authors:** Mohammad Nazeri, Jeffrey Watchorn, Sheldon Mei, Alex Zhang, Christine Allen, Frank Gu

**Affiliations:** a Institute of Biomedical Engineering, University of Toronto, 164 College St, Toronto, ON M5S 3G9, Canada; b Acceleration Consortium, 700 University Avenue, Toronto, ON M7A 2S4, Canada; c Department of Chemical Engineering and Applied Chemistry, University of Toronto, 200 College St, Toronto, ON M5S 3E5, Canada; d Department of Mechanical & Industrial Engineering, University of Toronto, 5 King’s College Rd, Toronto, ON M5S 3G8, Canada; e Leslie Dan Faculty of Pharmacy, University of Toronto, 144 College St, Toronto, ON M5S 3M2, Canada

**Keywords:** Real-Time Imaging Integration, Liquid Handling Robot, Automated Hydrogel Formulation, High-Throughput Material Characterization, Hardware Platform

## Abstract

In soft materials synthesis, rapid self-assembly and poor mechanical strength often limit the applicability of experimental characterization techniques. This limitation arises because transferring these materials to a suitable imaging platform is either too slow to capture the process of interest or impossible to safely transfer from the synthesis vessel to the characterization. In addition, the variable nature of these materials requires many experiments to understand the underlying structure–property relationships that govern these materials. In this work we present a new hardware platform that integrates simultaneous pipetting and in-situ imaging using the Opentron OT-2 liquid handling robot. A 3D printed adapter features two cylindrical openings, one containing the pipette tip to gantry adapter, and the other a USB camera. When the gantry picks up the pipette tip, the entire apparatus is lifted, allowing the camera to be used. This system enables real-time monitoring and characterization of dynamic processes, such as hydrogel crosslinking, without manual intervention. We used this system to characterize ionically crosslinked hydrogels, and monitored their properties over time, in a high-throughput and combinatorial manner. Although hydrogels were used as a proof-of-concept, this platform has broader applications in materials research, including crystallization dynamics, polymerization kinetics, and drug delivery system development.

## Graphical abstract

**Figure:**
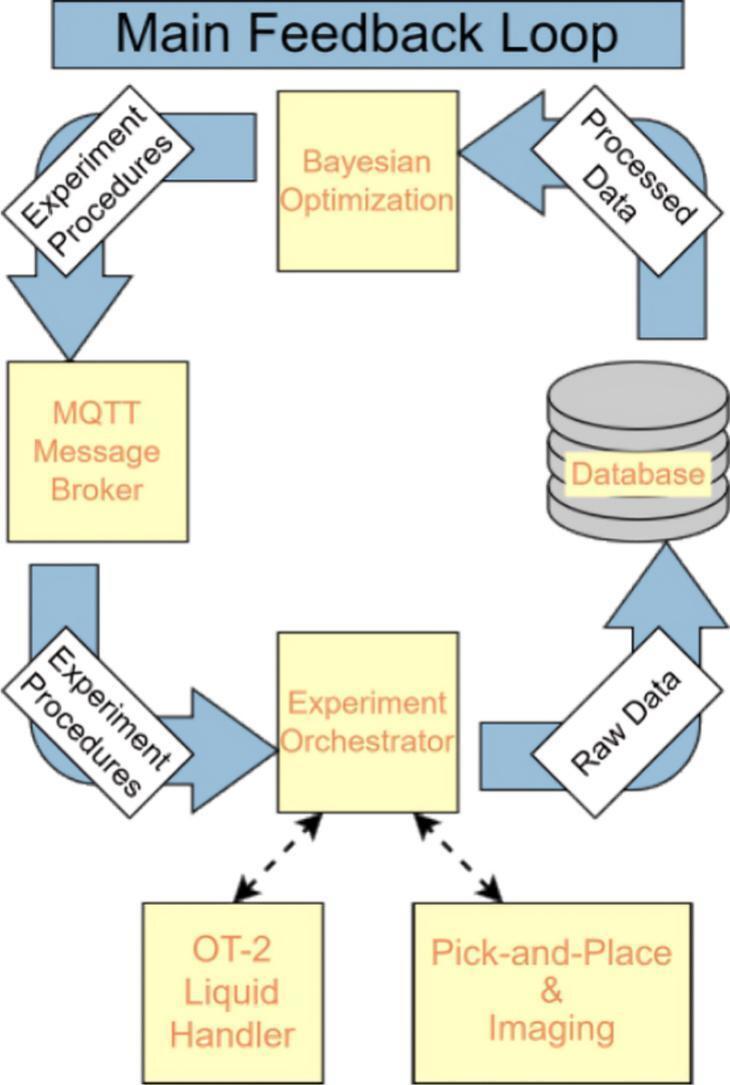

## 1 Specifications table

**Table:** 

Hardware name	Automated Hydrogel Imaging System
Subject area	• Engineering and materials science • Medical (e.g., pharmaceutical science)
Hardware type	• Imaging tools • Biological sample handling and preparation • Measuring physical properties and in-lab sensors
Closest commercial analog	No commercial analog is available
Open-source license	CC BY 4.0
Cost of hardware	$10,000 USD (OT-2) + $110 USD (Hardware Module)
Source file repository	https://doi.org/10.17632/xc5488grcv.3
Oshawa certification UID	CA000060

## 2 Hardware in context

Imaging during material synthesis is crucial for uncovering properties that are otherwise difficult to measure, such as reaction kinetics, structural changes, diffusion processes, and phase transitions [1], [2], [3], [4], [5]. This is particularly important for semi-solid or gel-like systems [6], [7], [8], where dynamic properties or insufficient mechanical strength make transferring samples to other instruments challenging [9]. Traditional imaging systems, such as microscopes, typically require moving samples from their synthesis location to the imaging system and involve significant labor to produce high quality images [10], [11]. Given that this labor is conducted by a human operator, there is significant variability in the accuracy and quality of imaging. This variability ultimately limits traditional imaging systems to the limits of the skill of the human operator, presenting as a bottleneck for accelerating materials discovery through laboratory automation.

To overcome these challenges, we developed an imaging platform that is compatible with commonly available automated liquid handling systems, in order to both streamline the sample preparation and characterization for sensitive soft materials. As a general platform we selected the Opentrons (OT-2) as it is both popular and open-source which makes the platform attractive to develop on to maximize uptake and usability by the broader scientific community [12], [13], [14], [15]. The modularity of open-source liquid handling systems provides a scalable approach to automation.

Several open-source platforms have demonstrated the advantages of modular automation in laboratory environments. For instance, OpenWorkstation introduced a concept where independent modules, such as pipetting and transport, can be integrated to create a versatile automated platform. Through the integration of modular components, including pipetting, transport, photo-crosslinking, and storage modules, the system improves lab workflow efficiency, decreases human error, and increases repeatability [16]. Similarly, Carvalho et al. present Osmar, an open-source, inexpensive substitute for commercial autosamplers used in liquid and gas sampling. Osmar provides accurate syringe manipulation with movement errors around 1 %, which is comparable to commercial versions, and is constructed utilizing modified low-cost CNC and 3D printing components [17]. Also, a study introduces BioCloneBot, a low-cost, 3D-printed liquid handling device that automates mixing, dispensing, and pipetting instructions. It is a versatile substitute for commercial handlers like as the Opentrons OT-2, providing researchers on a tight budget with great precision, repeatability, and customization [18]. Similarly, the MULA system provides a cost-effective and modular approach to liquid automation, designed for academic laboratories to automate chemical and biological tasks. Built using 3D-printed parts and a Hamilton gastight micro syringe, MULA offers high precision and reproducibility [19]. These examples illustrate the growing impact of open-source automation, which our imaging platform builds upon by integrating real-time imaging with automated liquid handling.

Imaging systems are widely used across various fields and industries [20], [21], [22]. As such, there has been significant interest in automating imaging systems for several applications, such as histology, drug delivery, and soft materials science [23], [24], [25], [26], [27]. Typically, these automated systems focus on autonomous image capture and processing across a sample area (such as a microscope slide). To expand the parallelism of these example systems, some more traditional gantry-based image capturing solutions were developed, to enable capturing of individual sample images. These traditional examples do not support dynamic experimentation (i.e., the addition of solvent or other materials to the sample stage) as they typically compromise liquid handling capacity in favor of imaging [28], [29]. There has been recent efforts to address this challenge in dynamic experimentation, for example Dunn et al. [30] reported a tool-changing approach leveraging the Science Jubilee platform which allows modular switching between fluid handling and imaging, however, since the same system handles fabrication, liquid dispensing, and imaging. In another study, Quinn et al [31] reported the PANDA platform, a self-driving lab for studying electrodeposited polymer films. The hardware is built on a modified CNC router with a custom 3D-printed tool holder replacing the spindle. This holder accommodates an electrochemical cell with two electrodes, a pipette tip connected to a syringe pump, and a telecentric lens with a camera for imaging [31]. These are both elegant solutions to the problem of enabling dynamic experimentation and imaging, but also both increase the complexity of the mechanical design and add new potential modes of failure to the gantry system during the tool changing steps (i.e. electromechanical failure during tool changing operations in the case of the Jubilee or relative misalignment and mechanical interference with the PANDA system). The motivation for this work was to expand the functionality of gantry type pipetting robots while leveraging the relatively simple and robust nature of friction fitting as a tool changing modality that is naturally designed within these systems. As such we present in this work a high-throughput automated image capture and analysis platform that leverages friction fitting to perform tool changes. The design enables continued experimentation by maintaining pipetting ability while orienting the camera in multiple different possible geometries.

## 3 Hardware description

The hardware platform developed for this study is a custom, solution designed to enable dynamic experiment monitoring, via imaging, during material synthesis. This is enabled by a 3D printed adapter that can exchange pipette tips while holding a vertically mounted camera. We leveraged a simple commercially available USB camera in these examples, but the design could be easily modified for any type of compact imaging solution, such as Pi cameras, provided they are able to be powered via the USB power headers within the Opentron platform. The tool is attached to the pipetting gantry with the same commands that are used natively to pick up pipette tips from a typical pipette tip holder. The entire apparatus remains attached to the pipetting gantry via friction fitting, which is able to transport both the adapter and any aspirated solution within the pipette tip. Generally, this friction-fitting type connection is able to carry a reasonable amount of weight and simplifies the process of attaching and detaching the adapter.

### 3.1 Component selection

The primary components of the hardware platform are as follows:

**Robotic pipetting gantry:** The pipetting gantry is based on an Opentron OT-2 liquid handling robot. This robot can be controlled by designing the experiment either using the Opentrons Protocol Designer or the Opentrons Python API, which enable no code or low code automation of liquid handling tasks, including aspirating, dispensing and mixing.

**Imaging System:** The imaging system is based on an Opti-Tekscope OT-HD USB camera. The camera is controlled via a custom instrument Class in the custom orchestration software. The Class enables the device to communicate with the OT-2 via simple serialized data structures using Python’s Socket library.

**Camera and pipette adapter:** The current form of this hardware uses a pipette tip and a vertical camera mount. This adaptor enables the camera to be in a vertical position and image directly beneath the gantry system. The hardware positioning can be modified to enable the camera to take images from different angles, which would be adaptable for a wider range of use cases. The distance between the camera and samples in a 96-well microplate on the OT-2 deck can be adjusted via adjusting the camera position or the length of the holder apparatus.

Several considerations were made to ensure that the holder would enable accurate and repeatable imaging. Firstly, the friction fitting that enables picking up the camera holder was attempted with multiple different mating designs. Ultimately, the final design presented in this work enables the greatest stability of imaging at the expense of the precision required to fit the holder to the pipette. Also, the design ensures that no collisions occur within the Opentrons deck, this was done to simplify the design, limiting the requirements for engineering controls.

### 3.2 Advantages of the hardware platform

The hardware platform offers several advantages:

#### 3.2.1 Real-time analysis

This feature reduces the possibility of errors resulting from post-processing analysis and can enable interventions to handle issues with the physical experimental setup (such as dynamic detection of collisions). Given that the imaging can be performed in real-time and in situ, one additional benefit is the immediate nature of the data collection, which helps to observe structural changes and material behavior as they occur. This approach is particularly valuable for capturing dynamic processes, such as the swelling, cross-linking, and phase transitions that occur in systems like hydrogels, where changes can happen rapidly and are not captured with post-synthesis analysis.

#### 3.2.2 High-throughput screening

**Fig. 1:**
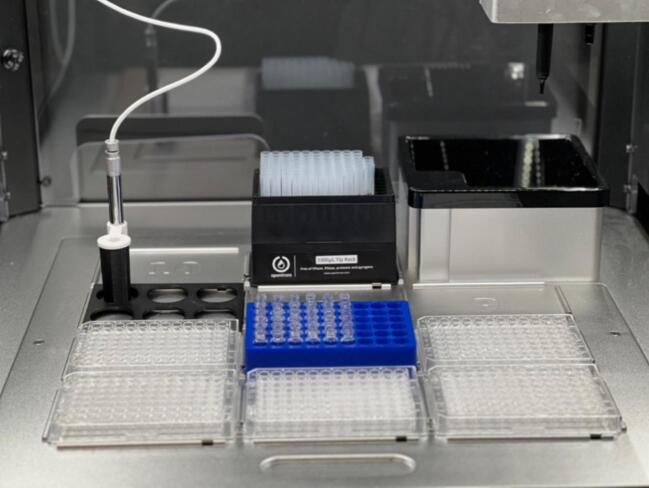
Deck configuration showing the high-throughput screening capability within the OT-2 robot.

High-throughput screening is desirable for accelerating discovery and optimization across a range of materials. The ability to simultaneously prepare and analyze formulations from multiple combinations of experimental parameters allows exploring vast formulation spaces efficiently, particularly in the development of complex systems like polymers, biomaterials, and drug delivery vehicles [32], [33]. This custom hardware platform, integrated into the OT-2 liquid handling robot, is designed specifically to facilitate such high-throughput experimentation. As shown in Fig. 1, the deck configuration accommodates up to six microplates, allowing for the monitoring of 576 unique combinations without human intervention. Additionally, the deck includes a 300 μL tip rack and a custom 3D-printed holder that can house 54 vials of 2 mL each. By systematically varying parameters and observing the gelation process, this platform significantly accelerates the discovery and optimization of hydrogel materials. This capability is particularly valuable for applications requiring extensive combinatorial testing, such as developing new biomaterials or optimizing cross-linking conditions for hydrogels.

This hardware platform is useful for more than just the immediate use case shown in this study. It makes real-time, automated characterization of dynamic laboratory processes possible by integrating imaging and sample preparation into a single integrated workflow. Researchers may find the platform especially helpful in:

• Real-time observation of dynamic processes, such chemical reactions or gelation, without interfering with the experimental setup.
• By combining imaging and sample preparation, manual handling and experimental variability were decreased.
• High-throughput, automated experimentation that makes it possible to effectively explore vast experimental design environments.
• Adaptability and affordability due to its open-source architecture and modular device compatibility.

https://doi.org/10.17632/xc5488grcv.3.

## 4 Design files summary

**Table:** 

Design file name	File type	Open-source license	Location of the file
Pick-and Place Apparatus	CAD file	CC BY 4.0	https://doi.org/10.17632/xc5488grcv.3
Pick-and-Place Holder	CAD file	CC BY 4.0	https://doi.org/10.17632/xc5488grcv.3
Deck Plate Adapter	CAD file	CC BY 4.0	https://doi.org/10.17632/xc5488grcv.3
2 mL Vials Holder	CAD file	CC BY 4.0	https://doi.org/10.17632/xc5488grcv.3
Tip Rack Height Booster	CAD file	CC BY 4.0	https://doi.org/10.17632/xc5488grcv.3

**Pick-and Place Apparatus (CAD file):** The file includes the 3D design of a pick-and-place apparatus that can carry a vertical camera in sync with the pipetting gantry's movement.

**Pick-and-Place Holder (CAD file):** The file contains the 3D design of a hollow cylinder extending upward to hold the pick-and-place apparatus.

**Deck Plate Adapter (CAD file**)**:** The file contains a rectangular plate, the same size as one of the Opentron decks, with six holes. Each hole is designed to fit a pick-and-place holder.

**2 mL Vials Holder (CAD file):** The file includes the 3D design of a holder specifically to securely position 2 mL vials during experimental procedures.

**Tip Rack Height Booster (CAD file):** The file includes the 3D design of a booster that fits under the tip rack to increase its height and ensure that there is no collision between the camera in its vertical position and any other labware, including vials.

## 5 Bill of materials summary

**Table:** 

Designator	Component	Number	Cost per unit −currency	Total cost − currency	Source of materials	Material type
Liquid Handler Robot	Opentron (OT-2)	1	$10,000	$10,000	LabX	Other
Camera	Opti-Tekscope OT-HD	1	$88.08	$88.08	Amazon	Metal
3D Printed Labware and Apparatus	3D printed PLA	1	$18.99	$18.99	Overture	Polymer
Screw	Super-Corrosion-Resistant 316 Stainless Steel Socket Head Screw	3	$0.72	$2.16	Amazon	Metal

## 6 Build instructions

The assembly begins with preparing the end cut in the Opentrons tip. A 1000uL Opentrons tip should be cut to the specified length (28 mm, Fig. 3 part 3), ensuring a clean and even cut enables good alignment during assembly. It is recommended to use a precision cutter or sharp blade to achieve accuracy. This shortened tip can be reserved for later, as it will be used in later steps in the assembly.

Next, the following objects need to be 3D printed, any material should be suitable, for the device shown in the later figures we printed all assemblies in polylactic acid (PLA):

### 6.1 Print the pick-and-place tool

**Fig. 2:**
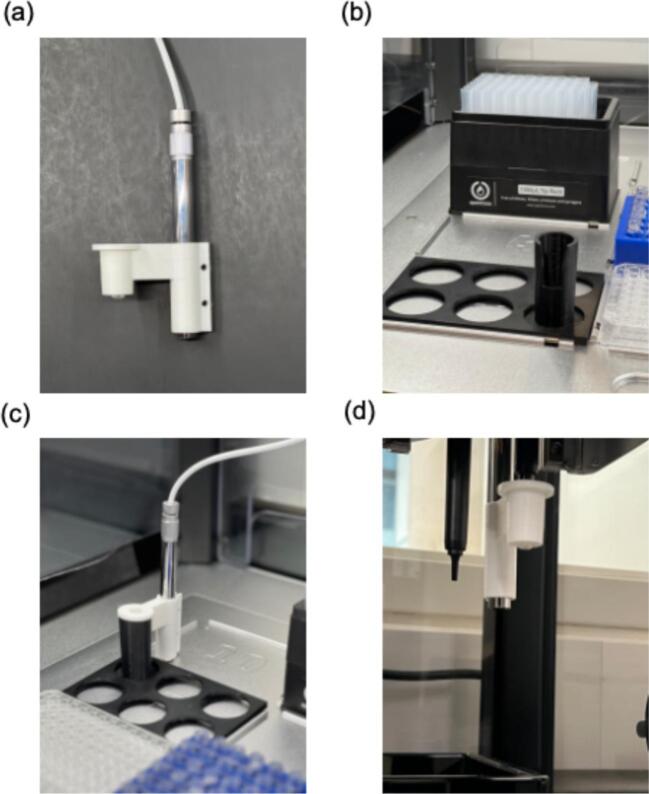
(a) A 3D model of the camera holder that contains the pipette tip holder and a cylindrical holder for positioning the camera. (b) The 3D-printed black PLA holder, designed to fit within one of the zones on the OT-2 deck, with a hollow vertical cylinder. (c) The camera module mounted in the holder on the OT-2 deck. (d) The pipette gantry lifting the assembled camera module from the holder.

A 3D model of the pick-and-place tool was created using SolidWorks, as shown in Fig. 2(a). The design possesses two openings parallel to each other. The smaller opening is designed as a pipette tip holder for the shortened 1000 µL tip, allowing the pipette gantry to pick up the entire 3D-printed apparatus without requiring additional modification to the pipettor. The larger opening created by the horizontal cylinder parallel to the pipette tip holder, affixes the camera to the rest of the assembly. The camera holder is additionally designed to maintain the camera at the appropriate angle for capturing real-time imaging during material synthesis on well plates or other labware, with our specific application focusing on visualizing hydrogel beads inside each well on 96-well microplate. Two screw holes are present in the camera holder in the current iteration of the design. These screw holes in conjunction with screws and nuts can be used to tighten the camera holder around the camera. Tightening the holder mitigates any small deviations in camera position as a result of vibrations through the camera mounting as the gantry operates to translate the position of the camera to the destination to be imaged.

Once the design was finalized, the 3D model was exported as an STL file and loaded into the Cura slicing software, compatible with the Prusa i3 Mk3s printer. Post-processing involved removing support structures and sanding any rough edges.

### 6.2 Assemble and calibrate the pick-and-place tool

**Fig. 3:**
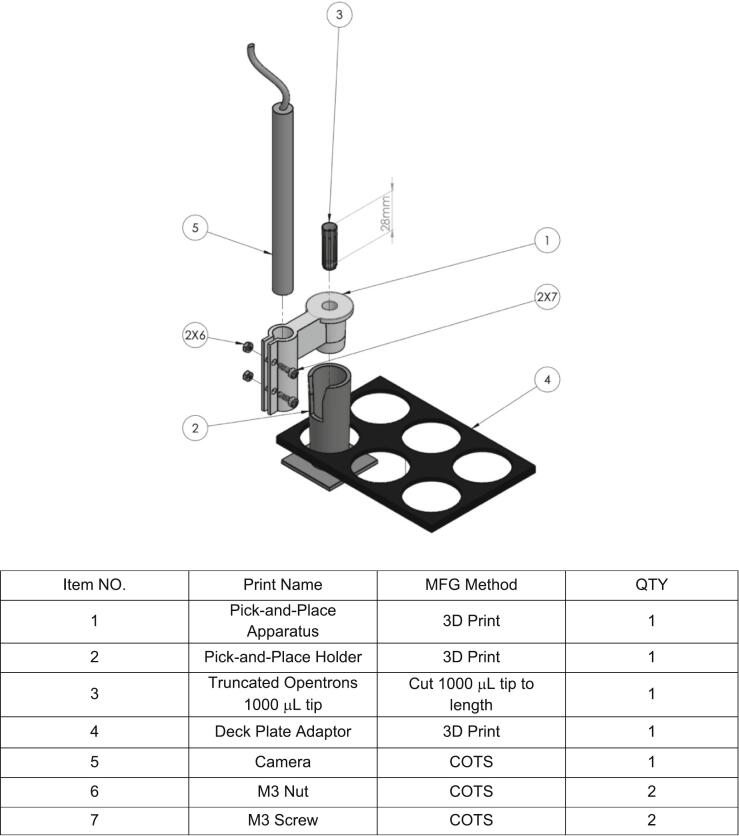
Exploded view of the Pick-and-Place apparatus with key components, manufacturing methods, and quantities. The design integrates 3D-printed parts, a truncated Opentrons tip, and COTS components for modular assembly.

Once the pick-and-place tool is printed, it can be easily assembled in a few steps. First, the shortened pipette tip prepared earlier can be inserted into the smaller opening of the pick-and-place tool with medium force. The tip should be inserted such that the cut end is facing down and into the assembly. Next the camera can be inserted into the larger opening, where the lens of the camera should be pointing downward, in the same direction as the shortened pipette tip. The camera should be snug within the holder and not require screws during assembling. At this stage, the camera can be inserted into the holder at a depth where approximately 10 mm of the camera protrudes from the bottom of the holder (Fig. 2(a)). As a rule of thumb, the camera should be inserted to a depth where it is the minimal amount of protrusion to limit any potential collisions with other labware. Any small changes in the vertical axis can be compensated for during the focusing and calibration of the device. For applications requiring a camera that is positioned further down within the holder, this type of action is supported by the design, but will need additional testing by the operator to ensure no mechanical interference between labware. Once the camera is coarsely positioned, the M3 screws and nuts can be threaded through the screw holes and then be tightened by hand to secure the camera in place in the holder. It should be noted that it is important to tighten the screws evenly. If the screws are not equally tightened, the camera may tilt slightly and cause a misalignment from the desired vertical position, which can affect the imaging accuracy of the system. A complete exploded visualization of the assemblies to aid in completing this section is detailed in Fig. 3. A video of the assembly process has been submitted as Supplementary Information.

Coarse calibration was then performed by adjusting the camera focus to achieve the clear images of the hydrogel beads. The camera can be focused by rotating the adjustable ring located at the top of the camera. This focusing was conducted manually and visually using the typical webcam app on our orchestrating computer, with a live feed open. Initial tests included capturing images of empty wells to validate the accuracy and stability of the setup.

### 6.3 Print the deck holder for the pick-and-place tool

A separate 3D-printed piece needs to be fitted into one of the zones on the deck, where the pick-and-place tool can be placed when not in use. As shown in Fig. 2(b), the pick-and-place holder consists of two components designed for use on the OT-2 deck. The Pick-and-Place Holder is a vertical hollow cylinder designed to hold the pick-and-place apparatus firmly, which ensures that the pipetting tool can be removed and picked up repeatably by the OT-2 pipette. The Deck Plate Adapter is a rectangular plate, the same size as one of the Opentron deck zones (∼128 mm x 85 mm), and has six evenly spaced holes, each meant to hold a pick-and-place holder. The pick and place holder enables the pick-and-place tool to be affixed to and ejected from the pipette in the same manner as a standard pipette tip. Briefly, the deck holder acts akin a pipette tip rack, which resists the compression from the pipette as is it lowered onto the mating surface of the pick-and-place tool. The force applied by the z-axis linear actuator in the OT-2 enables the friction fitting of the pick-and-place tool, where the widening top opening of the deck holder enables the pick-and-place tool to be removed freely from the deck slot that contains the deck holder. The pick-and-place tool can be similarly returned to the deck holder by leveraging the pipette tip ejector mechanism of the OT-2 pipette. This mechanism is a a concentric cylinder surrounding the pipette tip that actuates parallel to the shaft and pushes any pipette tip-type accessory free of the pipette. Thus, to return the pick-and-place tool to the original deck location, all that is required is to return the gantry to the deck holder location and eject the tip using the Opentrons API drop tip function. This reuse of the existing factory defined protocols as well as designing these tools to work in the same way as a traditional pipette tip translates the precision of liquid handling gantries to tool-based actions without requiring additional calibration. Generally, this type of approach could be applied with any system that possesses a concentric ejector design and is not solely limited to OT-2 pipettes.

### 6.4 Integrate the camera module with the OT-2

The camera module connects to a Python socket server via USB. The USB cable was secured by taping it to the moving gantry of the OT-2, keeping it suspended above the labware on the deck to prevent tangling during gantry movement. Once the camera module is connected, the control server should be downloaded to the computer using Git and your IDE of choice. The server runs a Python-based program that is responsible for controlling operations of the OT-2 and data acquisition from the camera module. The server also wraps the OpenCV-Python library, a free and open-source computer vision tool, to read image data from the camera module. Using OpenCV, the camera can capture raw RGB images with 640x480 pixel resolution. In addition to OpenCV, Pillow and Tkinter were additionally integrated into the server instance, to display a live feed image from the camera during operation. Once the code is executed, the pipette gantry will move to the designated zone, pick up a tip that is tightly fitted into one of the cylinders on the pick-and-place apparatus (as shown in Fig. 2(c)), and lift the entire assembly that includes the camera. The assembly of the camera into the holder and the integration of the entire setup into the OT-2 liquid handler are demonstrated in Fig. 2(d).

## 7 Operation instructions

### 7.1 Initial setup of the OT-2

**Fig. 4:**
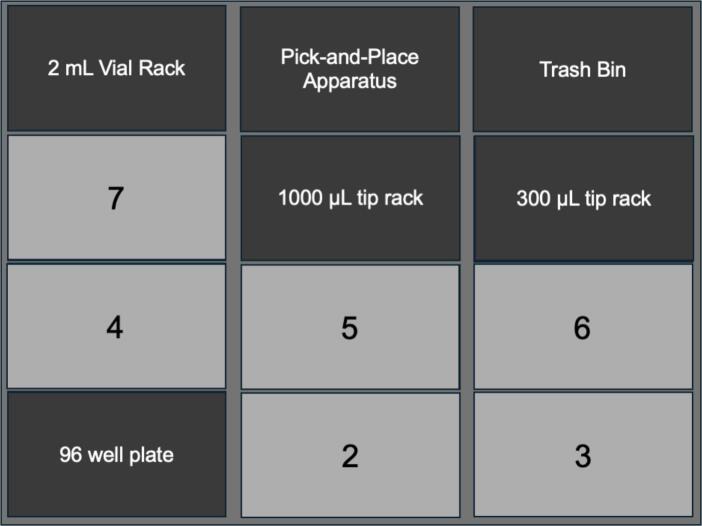
OT-2 deck layout showing labware and tool positions.

To prepare the OT-2 liquid handler for operation, we begin by setting up the deck. The components required to perform the example protocol are: the 3D printed pick-and-place apparatus, the camera holder, one 1000uL pipette tip rack and tips, one 300uL pipette tip rack and tips, one 96 well plate, and the 2 ml vial rack with vials containing solutions of interest. The OT-2 deck can then be populated with the necessary hardware in the following locations: 1–96 well plate, 8–1000 uL tip rack and tips, 9–300 uL tip rack and tips, 10–2 mL vial rack and vials, 11–camera holder with pick and place tool. If a user would like to change the positioning of these components for future studies, they may do so, as long as the locations are updated in the labware position definitions at the start of the Python protocol file. Once the labware is set up in the OT-2 deck it is critical to ensure that everything is secure and properly aligned to avoid any issues during the run. This can be tested by pressing down gently on any labware in a deck slot and listening for an audible click as the labware is slotted into the deck location. Next, the pipettes need to be attached to the OT-2 gantry. To run the example protocol a 1000 µl pipette should be affixed to the right mounting point in the OT-2 pipette carriage, along with a 300 µl pipette in the left mounting point. In the example provided, the OT-2 operates each of the two pipettes with distinct actions: One pipette is responsible for standard liquid-handling operations, such as picking up and dropping tips, as well as transferring, distributing, aspirating, and dispensing liquids. The second pipette is designed to interact with the 3D-printed pick-and-place apparatus. Once the tools, pipettes, and solutions are in place, the OT-2 can be turned on and connected to the computer via ethernet or wi-fi. The computer and OT-2 must be on the same local area network (LAN) and must be able to exchange data between themselves. The computer not only acts as a user interface to interact with the OT-2, but it is used as a camera control server (Section 7.3). After turning on and connecting the OT-2 it recommended to launch the Opentrons App and ensure that the OT-2 is visible and connected on the app dashboard prior to any test runs. Once the connection is confirmed, the calibration of the OT-2 and the camera device can be conducted. The labware position details on the OT-2 deck are shown in Fig. 4.

### 7.2 Calibration of the OT-2 and the camera position

The first step in calibrating the integrated OT-2 devices is to perform the factory standard calibrations on the OT-2. This procedure begins with the deck calibration. This calibration is conducted through the Opentrons App by selecting the deck calibration option in the settings menu and then following the on-screen instructions. The calibration consists of aligning the pipette tip over predefined positions on the OT-2 deck and manually jogging the pipettes into the correct locations. Once the deck calibration is complete, then the camera pick and place device can be calibrated. The camera calibration involves adjusting the camera lens focus by rotating the adjustable ring at the base of the camera (opposite the end with the camera lens and aperture). This calibration is done manually using a camera application on the computer that is connected to the camera. First, open the camera application, or any application that opens a live camera feed, and ensure that the camera is outputting video. Next, run the labware position check in the Opentrons app, following the on screen prompts. The labware definitions for the wells are defined such that they account for the height of the pick and place tool natively, limiting the potential of any collisions. While following the calibration workflow the camera device will be friction fit to the pipette and moved to over the 96 well plate at location 1. Once the camera is at the desired location, set the focus of the camera using the adjustable ring as described above. After the focus of the camera is set, the alignment of the camera can then be set by adjusting the offsets in the Opentrons app, to ensure that the camera is centered on the well. Once calibration is complete, we suggest executing the provided test script (Hydrogel Screening Testing Using Pick-and-Place Apparatus) to verify that the pick-and-place tool is correctly retrieved by the pipette gantry and that the camera accurately captures images of the wells. Once this step is complete, the settings are then saved in either the Opentrons App or as a configuration file (Labware Positions.json) uploaded in Mendeley dataset (https://doi.org/10.17632/xc5488grcv.3), minimizing the need for recalibration in future experiments. While the initial calibration of new pipettes is only required once, we recommend calibrating the labware on the deck before each experiment. This step ensures the precise positioning of both fluid dispensing and imaging, as we also discuss in the validation section regarding the importance of accurate positioning for creating hydrogel beads at the center of the wells.

### 7.3 Setting up the camera server

The computer that is connected to the OT-2 requires additional external Python packages to act as a camera control server. The packages should be installed in a Python virtual environment with the latest version Python 3.10 or above. OpenCV-Python is a Python package required to set up the camera and capture images. Pillow and Tkinter (built-in package) are used to create a custom graphical user interface that displays the live feed from the camera. The Python script *CameraServer.py* is responsible for capturing and saving images in the same folder every time that it receives a message from the OT-2. The IP address of the camera server on the LAN must be entered in *CameraServer.py* on line 155. Once ensuring that the Camera Server and OT-2 are connected on the same LAN, the automated liquid dispensing and imaging protocol can be executed. The *CameraServer.py* script can be executed directly in an integrated development environment (IDE), such as VS Code, with the appropriate Python environment selected. Alternatively, the script can be run manually by opening a terminal, navigating to the location where *CameraServer.py* is saved, activating the virtual Python environment, and entering *Python* *CameraServer.py* (Windows) or *Python3* *CameraServer.py* (Linux/MacOS). Once the script is running, a window showing a live feed from the camera will appear. Using this live feed verify that the camera is still in focus, and manually adjust the focus if necessary, following the same procedure as outlined in Section 7.2.

### 7.4 Execute imaging protocol

The hardware is operated by modifying and then executing “*Hydrogel Screening Testing Using Pick-and-Place* *Apparatus.py*”. The script defines the experimental setup, including labware configurations, pipette movements, and camera integration. A 96-well plate and a custom hardware rack are specified for this protocol. As the positions and movements are hard-coded in the example script, if the setup is modified from the demo, the following variable names would need to be updated prior to use: well_plate, reservoir_2ml, camera_tip, tips_1000 μl, and tips_300 μl for labware definitions (load_name and location), and right_pipette and left_pipette for pipette configurations (instrument_name, mount, and tip_racks). For demonstration purposes we have included a detailed video demo of the example protocol uploaded to Mendeley dataset (https://doi.org/10.17632/xc5488grcv.3). Briefly, the protocol starts with a 1000 µL pipette picking up a tip from the tip rack on slot 8. The pipette aspirates a solution of calcium chloride salt (CaCl_2_) from the 2 mL vial holder located at slot 10 and dispenses it into the first three wells (A1, A2, A3) of the 96-well plate positioned on slot 1. After dispensing, the pipette drops off its tip, and a 20 µL pipette picks up a tip from the tip rack on slot 9. Next, the 1000 µL pipette retrieves the 3D-printed pick-and-place apparatus with the attached camera from slot 11. Meanwhile, the 20 µL pipette aspirates three separate droplets of alginate solution, with air gaps in between to ensure that the droplets remain separated inside the tip for precise transfer. The pipette moves to the same well plate where the salt solution was distributed and begins dispensing droplets into the first well (A1). To ensure accurate dispensing with no loss of liquid, the dispensing step is split into two sub-steps. First the droplet is dispensed to just before the point of droplet detachment. Then the droplet is lowered by the pipette to touch the surface of the liquid in the well plate, where the droplet is then incorporated into the parent solution via coalescence. This method ensures the formation of clear and uniform hydrogel beads for the test system. It should be noted that this method may not be necessary for all materials systems of interest and could be modified to dispense directly into the well plate for the purposes of expediting the protocol if necessary. After the first droplet is dispensed, the camera is positioned above the first well (A1) to capture an image. The process then repeats for the rest of the wells specified in the control program. Before executing the protocol, the camera server must be instantiated on the control computer connected to the OT-2 as described in Section 7.3. Next, the IP address of the camera server must be replaced in the *Hydrogel_Screening_Testing_Using_Pick-and-Place_Apparatus.py* script (line 61). Next, to execute the protocol, go to the advanced robot settings on the OT-2 and launch a Jupyter Notebook. The Python script along with the additional custom labware definition files (*calab_8_tuberack_20000ul.json*, and *hardwarex_camera_rack.json*) must be uploaded to the same folder on the Jupyter Notebook. Finally, open a new terminal in the Jupyter Notebook and enter:

*Python3 /var/lib/jupyter/notebooks/Hydrogel_Screening_Testing_Using_Pick-and-Place_Apparatus.py*.

## 8 Validation and characterization

The custom hardware platform was validated and characterized for its performance in the high-throughput screening and analysis of alginate hydrogel formulations. Alginate is a natural biopolymer derived from brown seaweed and widely used in biomedical applications [34], [35] due to its ability to form gels in the presence of divalent cations such as calcium. Alginate gels are commonly used for drug delivery [36], tissue engineering [37], and as a thickening agent [38]. Clinically, alginate hydrogels are widely used in wound dressings [39] and have been explored in both preclinical and clinical trials for applications in drug delivery and cell encapsulation [40], [41]. The study focused on ionically crosslinked hydrogels formed through the interaction between alginate and CaCl_2_ solutions. While numerous studies have investigated alginate and CaCl_2_ hydrogels [42], [43], [44], this work uniquely combines automated hydrogel synthesis with real-time imaging of hydrogel beads, enabling in-situ characterization of the cross-linking process.

### 8.1 Detecting the effect of ion concentration on hydrogel beads

**Fig. 5:**
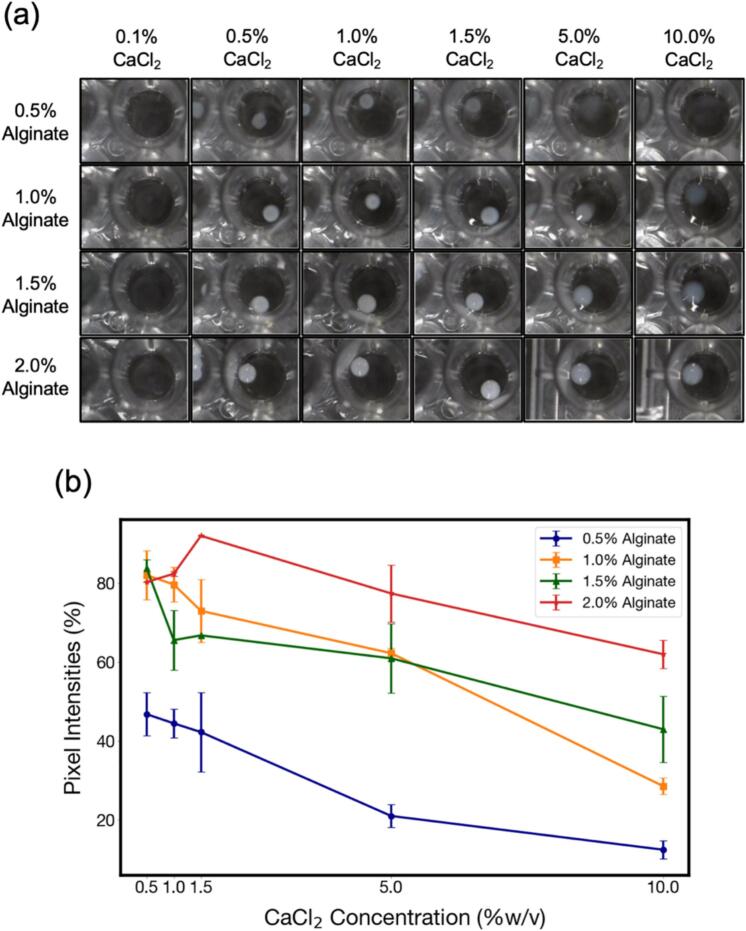
(a) Images of alginate beads formed at varying concentrations of alginate (0.5 %, 1.0 %, 1.5 %, 2.0 %) and CaCl_2_ (0.1 %, 0.5 %, 1.0 %, 1.5 %, 5.0 %, 10.0 %). (b) Pixel intensity analysis of the alginate beads that shows a decrease in intensity at higher CaCl_2_ concentrations, particularly beyond the critical R value, confirming reduced opacity and stiffness in the gels. The error bars represent the distribution of pixels intensity values.

We first tested the imaging system by observing the gel formation process and cross-linking efficiency of alginate hydrogels under varying concentrations of alginate and CaCl_2_. Specifically, the platform’s performance was assessed based on its ability to automatically prepare and monitor the gelation process across different concentrations of alginate (0.5 %, 1.0 %, 1.5 %, and 2.0 %) and CaCl_2_ (0.1 %, 0.5 %, 1.0 %, 1.5 %, 5.0 %, and 10.0 %). As shown in the Fig. 5(a), images were captured automatically 30 min after combining different concentrations.

At lower CaCl_2_ concentrations (0.1 %), the hydrogel beads were difficult to visualize. As the concentration increased, the beads became more opaque. However, when the concentration exceeded 1.5 %, reaching 5 % and 10 %, the beads became less opaque and more transparent. The observed opacity and transparency tied to numerical values of pixel intensity obtained through image processing. The images were analyzed by isolating gel beads within the images and measuring the pixel intensity values within the area corresponding to the beads. The gel beads were isolated using a combination of image enhancement and thresholding techniques to separate the bead from the background. Techniques such as histogram equalization were required to increase the contrast between more transparent beads and the background. The image processing produced a binary mask for isolating the area corresponding to the beads. Intensity values were measured on the scale of 8-bit integers (i.e. 0–255). Against a black background, areas of high pixel intensities are more opaque than areas of low pixel intensities. As shown in Fig. 5(b), image analysis confirmed these trends for different alginate concentrations, with a decrease in pixel intensity observed at higher CaCl_2_ concentrations. These observations align with previous publications concerning the critical value of calcium concentration for hydrogel formation [45], [46].

### 8.2 Monitoring hydrogel disintegration

**Fig. 6:**
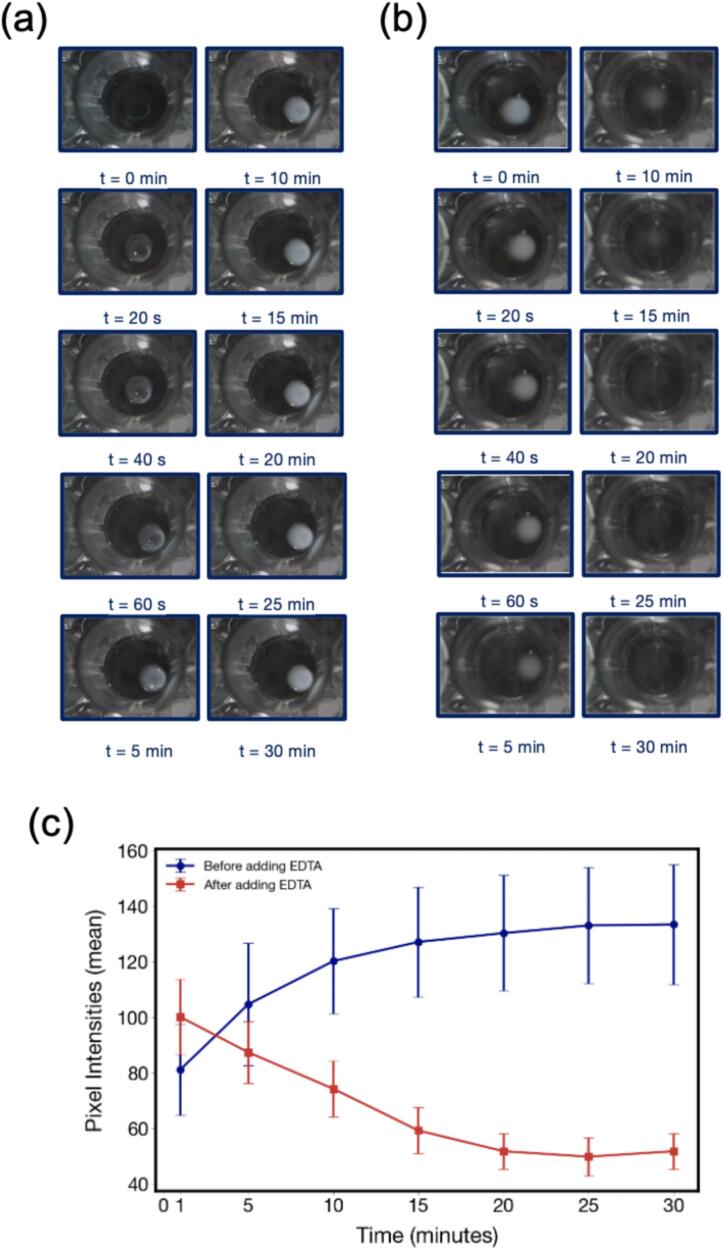
(a) Time-lapse images of hydrogels (1.5 % alginate, 1.0 % calcium) before EDTA addition, (b) Time-lapse images after 200 mM EDTA addition, showing progressive disintegration. (c) Mean pixel intensities over time, with stability before EDTA (blue) and rapid degradation after EDTA (red), reflecting the breakdown of calcium-alginate cross-links. (For interpretation of the references to colour in this figure legend, the reader is referred to the web version of this article.)

Next, we validated the hardware by monitoring the disintegration behavior of hydrogels composed of 1.5 % alginate and 1.0 % calcium in response to the addition of 200 mM ethylenediaminetetraacetic acid (EDTA). The mechanism of disintegration for these hydrogels in the presence of EDTA is caused by the chelating effect of EDTA, where calcium ions become trapped within EDTA and can no longer crosslink the alginate beads. The images captured at different time points before (Fig. 6(a)) and after the addition of EDTA (Fig. 6(b)) clearly show the gradual breakdown of the hydrogel structure, as indicated by the increased transparency of the beads over time. The imaging frequency per well depends on the gantry movement and positioning. Generally, the system can capture an image of a single well approximately every 5 s. For a full 96-well plate, the total imaging time varies based on the chosen intervals. For instance, if images are taken at intervals of 20 s, 40 s, 60 s, 5 min, 10 min, 15 min, 20 min, 25 min, and 30 min, completing the full plate scan takes approximately 3 h. This flexibility allows us to balance temporal resolution with overall throughput, ensuring effective monitoring of hydrogel formation and disintegration processes.

Quantitative analysis using pixel intensity measurements further corroborates these findings. As shown in Fig. 6(c), the graph compares the mean pixel intensities of the hydrogels over time before and after EDTA addition. Before EDTA addition, the pixel intensity steadily increases, reflecting the stable gel structure. After EDTA addition, however, a sharp decline in pixel intensity is observed, signifying disintegration of the hydrogel matrix. The rapid decrease in intensity within the first few minutes highlights the need for a system that can rapidly image these dynamic processes, which is addressed by this work.

## CRediT authorship contribution statement

**Mohammad Nazeri:** Writing – original draft, Visualization, Methodology, Investigation, Formal analysis, Data curation, Conceptualization. **Jeffrey Watchorn:** Writing – review & editing, Validation, Software, Methodology, Conceptualization. **Sheldon Mei:** Validation, Software, Methodology. **Alex Zhang:** Visualization, Software, Resources, Methodology. **Christine Allen:** Writing – review & editing. **Frank Gu:** Writing – review & editing, Supervision, Project administration, Funding acquisition, Conceptualization.

## Ethics approval

We confirmed that our work does not involve any animal or human experiments.

## Declaration of competing interest

The authors declare that they have no known competing financial interests or personal relationships that could have appeared to influence the work reported in this paper.
